# The influence of blue light on sleep, performance and wellbeing in young adults: A systematic review

**DOI:** 10.3389/fphys.2022.943108

**Published:** 2022-08-16

**Authors:** Marcia Ines Silvani, Robert Werder, Claudio Perret

**Affiliations:** ^1^ Faculty of Medicine, University of Lucerne, Lucerne, Switzerland; ^2^ Institute of Sports Medicine, Swiss Paraplegic Centre, Nottwil, Switzerland

**Keywords:** sleep quality, exercise, recovery, cognitive performance, physical activity

## Abstract

**Introduction:** Blue light from electronic devices has a bad reputation. It has a wavelength which may influence our circadian rhythm and cause bad sleep. But there are other aspects of blue light exposure which are often overlooked, for example, it may influence performance and wellbeing. However, few resources summarize its effects systematically. Therefore, the goal of this systematic review was to distil the present evidence on blue light exposure and its influence on sleep, performance and wellbeing and discuss its significance for athletes.

**Methods:** The databases that were searched were Cochrane, Embase, Pubmed, Scopus, and Virtual Health Library. The studies included investigated the influence of blue light exposure on either sleep, performance, wellbeing or a combination of those parameters on healthy humans. Quality assessment was done based on the quantitative assessment tool “QualSyst.”

**Results:** Summarizing the influence of blue light exposure, the following results were found (expressed as proportion to the number of studies investigating the particular parameter): Fifty percent of studies found tiredness to be decreased. One fifth of studies found sleep quality to be decreased and one third found sleep duration to be decreased. Half of the studies found sleep efficacy to be decreased and slightly less than half found sleep latency to be increased. More than one half of the studies found cognitive performance to be increased. Slightly more than two thirds found alertness to be increased and reaction time to be decreased. Slightly less than half of the studies found wellbeing to be increased.

**Conclusion:** Blue light exposure can positively affect cognitive performance, alertness, and reaction time. This might benefit sports reliant on team-work and decision-making and may help prevent injury. Blue light might also have negative effects such as the decrease in sleep quality and sleep duration, which might worsen an athlete’s physical and cognitive performance and recovery. Further research should explore if blue light can improve sleep, performance and wellbeing to significantly benefit athletic performance.

## 1 Introduction

Electronic devices, such as television, computers and smartphones have become permanent features of our everyday life. In combination with the increased use of those electronic devices a decrease in sleep quality has been reported (Hysing et al., 2015). This piqued researchers’ interest and it was found that blue light emitted by electronic devices suppresses the secretion of the hormone melatonin (Tordjman et al., 2017). One of the main functions of melatonin is the regulation of the circadian rhythm (Tordjman et al., 2017), which consequently influences sleep (Chang et al., 2015). The general consensus was that f blue light from electronic media negatively affects sleep quality. However, this is not a fair representation of the whole research that has been conducted concerning blue light. In fact, numerous studies report that blue light exposure did not only have negative, but also positive effects. For instance, it was reported that blue light exposure is an effective treatment against major depression symptoms (Strong et al., 2009), has a stimulating effect on cognitive brain activity (Vandewalle et al., 2013) and increases physical performance (Knaier et al., 2017b). The positive and negative effects of blue light are of interest for athletes for three reasons. Firstly, good sleep hygiene is the foundation of a strong performance (Samuels et al., 2016), it is therefore important to find out if sleep is negatively influenced by blue light. Secondly, many athletes suffer from sleep deprivation due to busy training schedules (Romyn et al., 2016), it is hence of interest to investigate if blue light exposure may improve performance by increasing alertness or cognitive function. Thirdly, wellbeing has an impact on athletic performance (Lastella et al., 2014) and thus it is out of interest whether this might be influenced by blue light exposure. Even though a vast amount of research has been conducted, a systematic analysis of existing findings is yet to be conducted, leaving the current standard of knowledge on blue light exposure unknown and the three questions mentioned above unanswered. This provided the rationale to conducting a systematic review on the influence of blue light on sleep, performance and wellbeing. In a first step, we decided to focus on healthy humans to ensure that enough data can be gathered for meaningful statements. At present, existing systematic reviews investigate the influence of blue light exposure on circadian rhythm (Tähkämö et al., 2019), macular health (Lawrenson et al., 2017), mental disorders (Srisurapanont et al., 2021) or tumors (Lai and Yew, 2016). To the best of our knowledge no systematic review has yet investigated the influence of blue light on sleep, performance and wellbeing either in elite athletes or in healthy humans. Therefore, the present study aimed to collect data to give clear and systematic insights on the current findings concerning these topics. The outcome of those findings will determine whether further studies are needed and if yes, what those studies might investigate.

## 2 Methods

This systematic review was conducted by following the PRSIMA (Preferred Reporting Items for Systematic Reviews and Meta-Analyses) guidelines (Moher et al., 2015).

### 2.1 Eligibility criteria

Randomized controlled trials, cohort studies, case-control studies and cross-sectional studies in English were reviewed. These studies had to investigate the influence of blue light exposure on either sleep, performance, wellbeing or a combination of those parameters. Studies that investigated the influence of blue light on participants with health issues (e.g., eye sickness, mental disorders or sleep-wake disorders) or explored only circadian phase and melatonin levels were excluded.

### 2.2 Source of information and search strategies

The following databases were searched: Cochrane, Embase, Pubmed, Scopus, and Virtual Health Library. The search strategy was a compound formed by the four cluster terms blue light, sleep, performance and wellbeing, connected to each other by the term “AND.” Terms related closely to the four cluster terms were connected to the latter by the term “OR.” To gather more data the search strategy was adjusted to only include three cluster terms, either “blue light, sleep and performance” or “blue light, sleep, and wellbeing.” A detailed overview of the search strategy is shown in Table 1. The search was conducted on 27th of September 2020.

**TABLE 1:** Overview concerning the search strategy.

Keywords	Cochrane		Embase		PubMed		Scopus		VHL	
	Hits	Selected studies	Hits	Selected studies	Hits	Selected studies	Hits	Selected studies	Hits	Selected studies
(1) Blue light exposure OR blue light	3	N/A	11,447	N/A	N/A	N/A	23,612	N/A	N/A	N/A
(2) Bedtime OR sleep quality OR recovery OR nighttime OR screen time	533	N/A	700287	N/A	260868	N/A	1205332	N/A	N/A	N/A
(3) Performance OR exercise OR sport OR physical activity	3,569	N/A	2358966	N/A	2135923	N/A	6714080	N/A	N/A	N/A
(4) Subjective wellbeing OR wellbeing OR mood	504	N/A	303592	N/A	451906	N/A	224331	N/A	N/A	N/A
Combined keywords										
(1) AND (2)	87	N/A	467	N/A	92	7	485	N/A	N/A	N/A
(1) AND (3)	3	N/A	757	N/A	118	6	2,160	N/A	N/A	N/A
(1) AND (4)	49	N/A	134	N/A	48	4	92	N/A	N/A	N/A
(1) AND (2) AND (3)	42	0	77	2	29	N/A	67	8	9	0
(1) AND (2) AND (3) AND (4)	8	0	19	1	7	N/A	8	0	N/A	N/A

**FIGURE 1:**
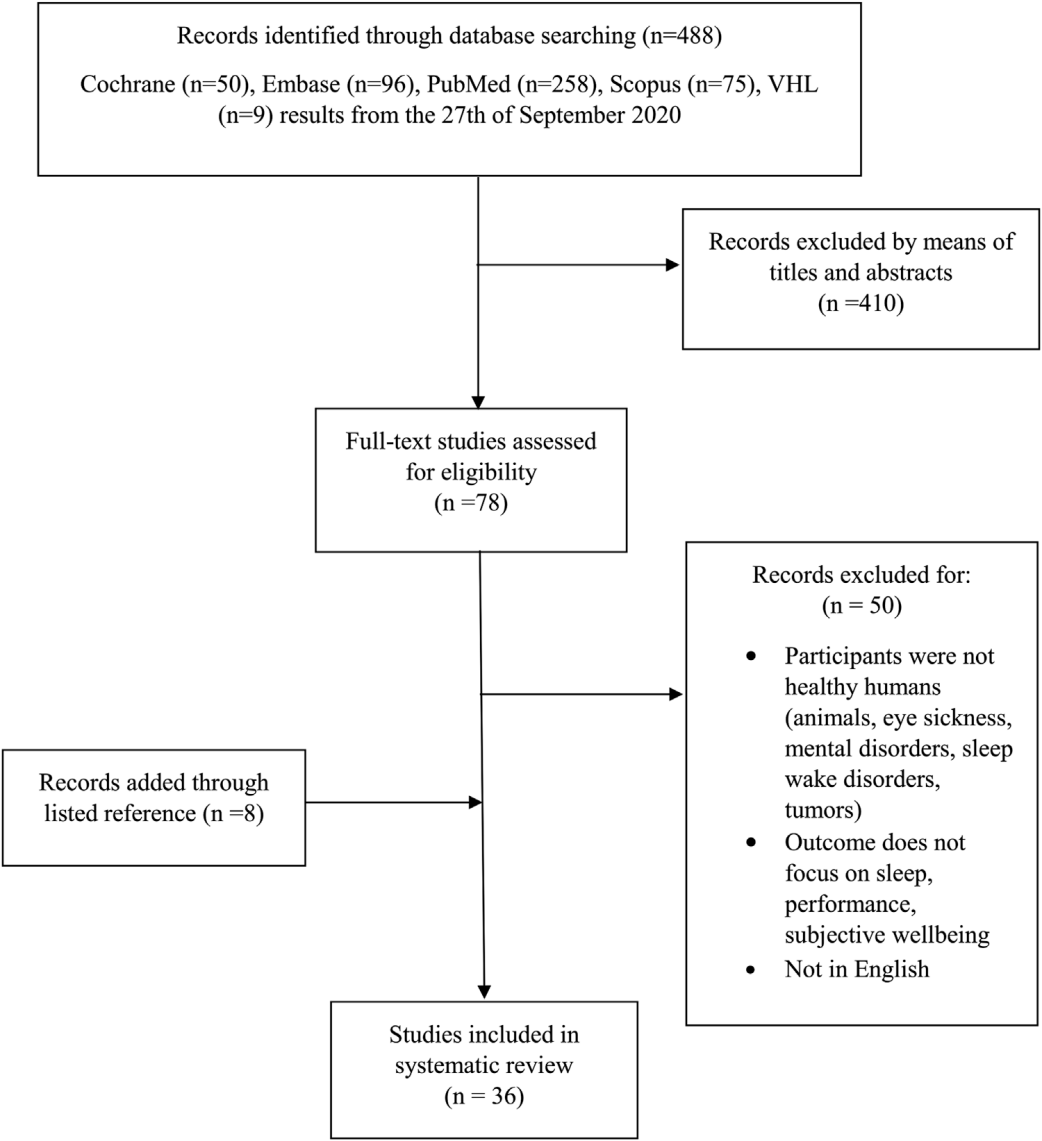
Selection process for research articles included in the review. Modified version from the recommendation in the PRISMA (Preferred Reporting Items for Systematic Reviews and Meta-Analyses) statement Moher et al. (2015). Legend: Virtual Health Library (VHL).

Number of hits on keywords and combined keywords in Title/Abstract with advanced search for Cochrane, Embase, PubMed, Scopus and VHL. For PubMed the filter “human” was added. The eight additional studies from listed references of the included studies are not represented on Table 1. For more information see Figure 1. N/A, Not applicable; VHL, Virtual health library.

### 2.3 Study selection and data collection process

All duplicates were removed and the studies were screened for the eligibility criteria. In a first step, titles and abstracts were screened and unsuitable studies were removed. The remaining studies were screened for full text. Additionally, listed references of the included studies were also screened. Figure 1 presents the detailed selection process for the research studies. The data collection included the age of the participants, their activity, the intervention type, the duration of the intervention, measurements, study design and the outcome on either sleep, performance or wellbeing.

### 2.4 Quality assessment

The quantitative assessment tool “QualSyst” was used for the quality assessment (Kmet et al., 2004). The studies were scored using fourteen items. Each item was marked with either a yes = 2, partial = 1, no = 0, or N/A = not applicable. Items marked with N/A were excluded from the calculation. The final score was determined by summing up the total score across the relevant items, expressed as a percentage of the theoretical maximum Studies were categorized as strong quality (>75%), moderate quality (55%–75%) and weak quality (<55%). This quality assessment was performed by two reviewers (MS, CP). Study quality scores that differed between reviewers were discussed until consensus was found.

## 3 Results

### 3.1 Study selection

The initial search strategy resulted in a total of 488 hits. After title and abstract screening, 78 studies were selected for full text screening. The screening of the listed references resulted in eight additional studies. After the full text screening 36 eligible studies based on systematic use of inclusion and exclusion criteria were recorded. The quality assessment of the 36 selected studies revealed 24 studies to be of strong and twelve of moderate quality. Detailed results of the quality assessment are presented in Table 2.

**TABLE 2:** Quality assessment “QualSyst” according to Kmet et al. (2004).

Study	Question described	Appropriate study design	Appropriate participant selection	Characteristics described	Random allocation	Researchers blinded	Participants blinded	Outcome measures well defined and robust to bias	Sample size appropriate	Analytic methods well described	Estimate of variance reported	Controlled for confounding	Results reported in detail	Conclusion supported by results	Rating
Alkozei et al. (2016)	2	1	1	2	0	N/A	N/A	2	1	2	2	1	2	2	strong
An et al. (2009)	2	1	2	2	0	N/A	N/A	2	1	2	1	1	2	2	strong
Ayaki et al. (2016)	2	2	2	2	0	0	0	2	0	1	1	0	2	2	moderate
Baek and Min (2015)	2	2	1	2	0	N/A	N/A	2	1	2	2	1	2	2	strong
Beaven and Ekström (2013)	2	2	1	2	1	1	1	2	1	2	1	1	2	2	strong
Bowler and Bourke (2019)	2	2	2	1	1	0	N/A	1	2	2	2	1	2	2	strong
Burkhart and Phelps (2009)	2	2	2	2	2	1	N/A	2	1	2	2	1	2	2	strong
Cajochen et al. (2011)	2	2	2	2	0	0	2	2	0	2	1	1	2	2	moderate
Chang et al., (2015)	2	2	2	2	1	N/A	N/A	2	0	2	2	0	2	1	strong
Chellappa et al. (2013)	2	2	1	2	0	2	N/A	2	2	2	2	0	2	1	strong
Chindamo et al. (2019)	1	2	2	2	N/A	N/A	N/A	1	2	2	1	2	2	2	strong
Driller and Uiga (2019)	2	2	1	2	1	N/A	N/A	2	1	2	2	2	2	2	strong
Ekström and Beaven (2014)	2	2	2	2	1	1	2	1	1	2	2	0	2	1	strong
Gabel et al. (2013)	2	2	2	2	0	N/A	1	2	1	2	1	1	2	2	strong
Grønli et al. (2016)	2	2	0	2	2	2	N/A	2	1	2	1	1	2	1	strong
Figueiro et al. (2009)	2	2	1	2	0	N/A	N/A	2	1	2	2	1	2	2	strong
Heath et al. (2014)	2	2	2	2	0	0	0	2	0	2	1	2	2	1	moderate
Heo et al. (2017)	2	2	2	2	1	1	2	2	0	2	2	1	2	1	strong
Iskra-Golec et al. (2012)	2	2	2	2	0	0	0	1	1	2	0	1	2	1	moderate
Knaier et al. (2017a)	2	2	2	2	1	2	1	2	1	2	2	0	2	2	strong
Knufinke et al. (2018)	1	1	2	2	N/A	N/A	N/A	1	2	2	2	1	2	2	strong
Lehrl et al. (2007)	2	2	1	1	0	0	1	1	1	2	1	1	2	2	moderate
Lockley et al. (2006)	2	2	2	2	1	0	0	2	0	2	1	1	2	2	moderate
Motamedzadeh et al. (2017)	2	2	2	2	0	N/A	N/A	2	1	1	1	0	2	2	moderate
Münch et al. (2016)	2	2	2	2	1	N/A	1	2	0	2	2	1	2	2	strong
Phipps-Nelson et al. (2009)	2	1	2	2	0	N/A	2	2	1	2	1	0	2	1	moderate
Rångtell et al. (2016)	2	2	2	2	1	N/A	N/A	2	0	2	2	0	2	1	strong
Sahin and Figueiro (2013)	2	2	2	2	1	N/A	N/A	2	1	2	1	0	2	2	strong
Scheuermaier et al. (2018)	2	1	2	2	0	0	0	2	0	2	1	1	2	2	moderate
Slama et al. (2015)	2	2	2	2	1	0	N/A	2	0	2	1	1	2	2	moderate
Sander et al. (2015)	2	2	2	1	2	N/A	N/A	1	1	2	2	0	2	1	strong
Taillard et al. (2012)	2	2	2	2	2	2	2	2	1	2	2	0	2	2	strong
Tulppo et al. (2014)	2	2	2	2	1	2	2	2	0	2	1	1	2	1	strong
Van Der Lely et al. (2015)	2	2	2	2	0	N/A	N/A	2	1	2	2	1	2	2	strong
Viola et al. (2008)	2	2	1	2	0	0	2	1	2	2	2	1	1	2	moderate
Yang et al. (2018)	2	2	2	2	0	0	0	2	0	1	1	0	2	2	moderate

N/A not applicable, 2 indicates yes, 1 indicates partial, 0 indicates no; Quality scores: >75% strong, 55% ≥ 75% moderate, <55% weak.

### 3.2 Blue light and sleep

#### 3.2.1 Age, intervention, and duration

Twenty-four studies investigated the influence of blue light exposure on sleep (Table 3). The participants’ average age was 26 years, with one study not mentioning the exact age of their participants other than indicating that they were all adults (Rångtell et al., 2016). Twelve studies compared blue light with a different colored light intervention such as white, red or orange light (Table 3). Twelve studies used electronic devices such as smartphones, tablets and computers and compared them with blue light filter control conditions. Such as blue light blocking glasses or blue light filters for displays (Table 3). The average exposure time was 2.2 h. Those studies with longer exposure times were separately calculated and averaged around 2.75 weeks. One study did not mention the exact exposure time (Chindamo et al., 2019). Exact exposure times for each study are listed on Table 3.

**TABLE 3:** Effects of blue light on sleep.

Study	Age (years) participants (*n*)	Activity	Intervention/Exposure	Duration of intervention	Measurement tool	Methodological characteristics	Outcome
Ayaki et al. (2016)	29 ± 5	Tasks on portable device before bedtime	iPad without BLB glasses vs. iPad with BLB glasses	2 h	Actigraphy to monitor sleep, modified KSS and PSQI	Controlled study	Sleep efficacy significant ↑ with BLB glasses
	*n* = 12						Sleep latency significant ↓ with BLB glasses
							Sleepiness no significant ↓ in BL
Bowler and Bourke (2019)	20.5	Facebook before bedtime	iPad without BL filter vs. iPad with amber filter	22.5 min	Modified PSQI	RCT	Sleep quality no significant change in BL
	*n* = 30						
Burkhart and Phelps (2009)	34 ± 8.2	Bedtime routine	Yellow tinted safety glasses vs. BLB amber glasses	3 h	Sleep diary with Likert scale	RCT	Sleep quality significant ↑ with BLB glasses in the last week
	*n* = 20						
Cajochen et al. (2011)	23.8 ± 5.0	Watching a relaxing movie and reading tasks	BL computer screen, (6.953 K LED, 440–470 nm) vs. CCFL (4.775 K)	5 h	KDT, KSS	Controlled crossover study	Subjective sleepiness significant ↓ in BL
	*n* = 13						
Chang et al. (2015);	24.9 ± 2.9	Reading before bedtime	Tablet (452 nm) vs. hard copy book	4 h	EEG, KSS, PSG	RCT, crossover study	Evening sleepiness significant ↓ in BL
							Morning alertness significantly delayed in BL
	*n* = 12						Sleep efficiency, total sleep time no significant change in BL
							Sleep latency significant ↑ in BL
Chellappa et al. (2013)	25.2 ± 3.1	Sitting in the evening	BL (6.5 K) vs. WL (3 K)	2 h	All night EEG	Controlled crossover study	Wakefulness no significant ↓ in BL
	*n* = 30						
Chindamo et al. (2019)	17.5 months	Tasks on portable device during daytime routine	Tablets or smartphones vs. hard copy book	Everyday use	Questionnaire	Cross-sectional study	Sleep onset latency significant ↑ in BL
	*n* = 1,117						Total sleep time significant ↓ in BL
Driller and Uiga (2019)	28 ± 5.0	Reading before bedtime	iPad vs. hard copy book	1 h	Actigraphy, Likert scale	RCT, crossover study	Sleep duration and sleep efficiency no significant negative change in BL
	*n* = 14						Sleep quality no significant ↓ in BL
Gabel et al. (2013)	23.1 ± 0.8	Cognitive tasks during morning hours	BL (470 nm) vs. DSL	20 min	KSS	Controlled study	Subjective sleepiness significant ↑ in BL after second night
	*n* = 17						
Grønli et al. (2016)	25.1 ± 2.9	Reading before bedtime	iPad vs. hard copy book	30 min	KSS, Online questionnaire, PSG, sleep diary	RCT, crossover study	Sleep duration, sleep onset latency no significant change in BL
	*n* = 16						Subjective sleepiness significant ↓ in BL
Heath et al. (2014)	17.4 ± 1.9	Playing games before bedtime	iPad vs. iPad with shortwavelength filter	48 min	PSG for sleep onset latency, SSS	Counterbalanced controlled study	Sleep onset latency and subjective sleepiness no significant change in BL
	*n* = 16						
Heo et al. (2017)	31.0 ± 4.2	Smartphone use during the morning	Smartphone vs. Smartphone display filter	150 min	ESS, FSS, PSQI	RCT, crossover study	Sleepiness significant ↓ in BL
	*n* = 22						
Iskra-Golec et al. (2012)	28.3 ± 2.8	Sitting, office work during the day	BL (17 K, 420 nm–480 nm vs. WL (4 K)	3 weeks, office hours	KSS during week	Field experiment counterbalanced	Sleepiness significant ↑ in BL
	*n* = 30						
Knufinke et al. (2018)	18.8 ± 3.0	Elite athletes training	BL emitting activities within the last hour before bedtime	1 h	CSD, HSDQ, KSS, online survey, diaries, PSQI, SHI	Qualitative study	Sleep onset latency no significant ↓ in BL
	*n* = 98						
Lockley et al. (2006)	23.3 ± 2.4	Sitting still	BL (460 nm) vs. 555 nm-light	6.5 h	KSD, KSS during BL	RCT	Subjective sleepiness significant ↓ during BL, but not at onset of BL exposure
	*n* = 16						
Motamedzadeh et al. (2017)	30.2 ± 4.1	Sitting during night shift	BL (17 K) vs. BL (6.5 K) vs. WL	1 week	KSS	RCT	Sleepiness significant ↓ in BL
	*n* = 30						
Münch et al. (2016)	23.2 ± 3.3	Bedtime routine	BL (750 lux, 3,537 K) vs. OL (100 lux, 1,500 K) vs. CTRL (40 lux, 2,600 K)	30 min	EEG, VAS	RCT, Crossover study	Subjective sleepiness significant ↓ in BL compared to CTRL
	*n* = 18						Total sleep time significant ↓ in BL compared to OL
Phipps-Nelson et al. (2009)	32.1 ± 6.8	Driving during the night	BL (430 nm) vs. RL (620 nm)	6 h	KSS	RCT	Subjective sleepiness no significant change in BL
	*n* = 8						
Rångtell et al. (2016)	Adults	Reading a book	LED tablet (ASUS Transformer Pad TF700) vs. hard copy	2 h	KSS, PSG	RCT	Sleep duration, sleep onset latency, subjective arousal and subjective sleepiness no significant change in BL
	*n* = 14						
Sahin and Figueiro (2013)	20.8	Sitting, post lunch dip	BL (470 nm) vs. RL (630 nm)	48 min	KSS	RCT	Subjective sleepiness no significant change in BL
	*n* = 13						
Sander et al. (2015)	65	Spending time at home	BL (5100 K, 450 nm) vs. Blue-suppressed light (2800 K, 625 nm)	3 weeks	PSQI, questionnaire	RCT, crossover study	Sleep duration, sleep quality no significant change in BL
	*n* = 38						
Van Der Lely et al. (2015)	16	Sitting in the evening	CL glasses vs. BLB glasses	3 h	EEG, KSS	Balanced crossover study	Subjective sleepiness significant ↑ with BLB glasses
	*n* = 13						
Viola et al. (2008)	36.4 ± 10.2	Office work during the day	BL (420–480 nm) vs. WL	4 weeks	KSS, PSQI	Controlled crossover study	Daytime sleepiness significant ↓ in BL
							Evening fatigue significant ↓ in BL
	*n* = 94						Sleep duration significant ↑ in BL
							Sleep quality significant ↑ in BL
Yang et al. (2018)	20 ± 3.4	Nightshift work	Intermittent BL (6000 K) vs. continuous bright light vs. continuous dim light (3600 K)	30 min	KSS, PSG	RCT	Sleep efficiency significant ↓ in BL and in continuous bright light
	*n* = 15						Sleep onset latency no significant change in BL Total sleep time significant ↓ in BL and in continuous bright light

Increase (↑), decrease (↓), blue light (BL), blue light blocking (BLB), cold cathode fluorescent lamp (CCFL), clear lenses (CL), Consensus Sleep Diary (CSD), dawn simulation light (DSL), electroencephalography (EEG), Epworth Sleepiness Scale (ESS), Fatigue Severity Scale (FSS), Holland Sleep Disorder Questionnaire (HSDQ), thousand (K), Karolinska Drowsiness Test (KDT), Karolinska Sleep Diary (KSD), Karolinska Sleepiness Scale (KSS), light emitting diode (LED), orange light (OL), polysomnography (PSG), Pittsburgh Sleep Quality Index (PSQI), randomized controlled trial (RCT), red light (RL), Sleep Hygiene Index (SHI), Stanford Sleepiness Scale (SSS), Visual Analogue Scale (VAS) and white light (WL).

#### 3.2.2 Activity and measurement

Activity during the blue light exposure included relaxed sitting, leisure, bedtime routine, reading on an electronic device, mundane tasks on a smartphone such as Facebook use, cognitive tasks, driving a car, office work, and physical training (Table 3). The most commonly used methods to measure the influence of blue light on sleep were the Karolinska Sleepiness Scale (KSS), polysomnography (PSG) including electroencephalography (EEG), the Pittsburgh Sleep Quality Index (PSQI), questionnaires, actigraphy and Likert scale (Table 3). The following methods of measurement were used less frequently across the included studies: Consensus Sleep Diary (CSD), Epworth Sleepiness Scale (ESS), Fatigue Severity Scale (FSS), Holland Sleep Disorder Questionnaire (HSDQ), Karolinska Drowsiness Test (KDT), Karolinska Sleep Diary (KSD), Sleep Hygiene Index (SHI), Stanford Sleepiness Scale (SSS) and Visual Analogue Scale (VAS) (Table 3). The methods of measurements were applied either before, during or after the blue light intervention.

#### 3.2.3 Sleep responses to blue light

The term sleep included the elements tiredness, sleep quality, sleep duration, sleep efficacy, and sleep latency. More details to each element are given in the following sections.

##### 3.2.3.1 Tiredness

Tiredness can be assessed in the categories sleepiness, fatigue, wakefulness, alertness, and arousal. For sleepiness the following data was found: eight studies reported a decrease in subjective sleepiness when exposed to blue light conditions (Lockley et al., 2006; Viola et al., 2008; Cajochen et al., 2011; Chang et al., 2015; Grønli et al., 2016; Münch et al., 2016; Heo et al., 2017; Motamedzadeh et al., 2017). However, one study reported a higher subjective sleepiness while wearing blue light blocking glasses when compared to the blue light condition, where no blue light blocking glasses were worn (Van Der Lely et al., 2015). Five studies did not find any significant change in subjective sleepiness between blue light and non-blue light conditions (Phipps-Nelson et al., 2009; Sahin and Figueiro, 2013; Heath et al., 2014; Ayaki et al., 2016; Rångtell et al., 2016) and two studies reported an increase in subjective sleepiness when exposed to the blue light condition (Iskra-Golec et al., 2012; Gabel et al., 2013). One study found fatigue to be decreased following blue light exposure (Viola et al., 2008). One study found wakefulness not to be significantly decreased when exposed to the blue light condition (Chellappa et al., 2013), however one study found that morning alertness was delayed when exposed to the blue light condition, adding to the evidence that blue light may increase tiredness (Chang et al., 2015). Another study showed no significant change in arousal levels (Rångtell et al., 2016). Summarizing all these results under the term tiredness, seventeen studies reported measures related to tiredness. Out of those seventeen studies, nine studies reported blue light exposure to be decreasing tiredness. But because the term tiredness categorizes sleepiness, fatigue, wakefulness, morning alertness, and arousal, the studies which included more than one of these characteristics should be counted for the number of times mentioned. This means Chang et al. (2015), Rångtell et al. (2016), Viola et al. (2008) should be counted twice, creating a new total of 20 occurrences. Out of those 20 occurrences, ten occurrences, counting the reference Viola et al. (2008) twice, reported of decreasing tiredness (Lockley et al., 2006; Viola et al., 2008; Cajochen et al., 2011; Chang et al., 2015; Van Der Lely et al., 2015; Grønli et al., 2016; Münch et al., 2016; Heo et al., 2017; Motamedzadeh et al., 2017). Seven occurrences, with reference Rångtell et al. (2016) counting twice, reported no significant change between conditions (Phipps-Nelson et al., 2009; Chellappa et al., 2013; Sahin and Figueiro, 2013; Heath et al., 2014; Ayaki et al., 2016; Rångtell et al., 2016). And three studies reported an increase in tiredness in the blue light condition (Iskra-Golec et al., 2012; Gabel et al., 2013; Chang et al., 2015). Summarized, there were thirteen out of 20 occurrences which reported blue light to be influencing tiredness, out of which ten occurrences reported a decrease and three studies reported an increase in tiredness.

##### 3.2.3.2 Sleep quality

One study found that sleep quality was higher in the non-blue light condition compared to the blue light condition, adding to the evidence that blue light can decrease sleep quality (Burkhart and Phelps, 2009). Three studies reported nonsignificant changes in sleep quality by blue light exposure (Sander et al., 2015; Bowler and Bourke, 2019; Driller and Uiga, 2019). Additionally, one study reported increased sleep quality following the blue light condition (Viola et al., 2008). There was one (Burkhart and Phelps, 2009) out of five studies that suggested blue light to be decreasing sleep quality and one (Viola et al., 2008) out of five studies suggesting an increase in sleep quality through blue light exposure.

##### 3.2.3.3 Sleep duration

One study reported an increase in sleep duration when exposed to the blue light condition (Viola et al., 2008), however three studies reported a decrease in sleep duration when exposed to the blue light condition (Münch et al., 2016; Yang et al., 2018; Chindamo et al., 2019). Five studies showed no significant change in sleep duration (Chang et al., 2015; Sander et al., 2015; Grønli et al., 2016; Rångtell et al., 2016; Driller and Uiga, 2019). Summarizing these findings, three out of nine studies reported of decreasing sleep duration through blue light exposure and only one study reported an increase in sleep duration through blue light exposure.

##### 3.2.3.4 Sleep efficacy and sleep latency

Two studies showed a higher sleep efficacy in the non-blue light condition compared to the blue light condition, adding to the evidence that blue light exposure can decrease sleep efficacy (Ayaki et al., 2016; Yang et al., 2018) Two other studies showed no significant change in sleep efficacy following the blue light condition (Chang et al., 2015; Driller and Uiga, 2019). This means two out of four studies found sleep efficacy to be decreased following blue light exposure. Sleep latency was found to be decreased in the non-blue light condition compared to the blue light condition in one study, (Ayaki et al., 2016). Two other studies found an increase in sleep latency in the blue light condition (Chang et al., 2015; Chindamo et al., 2019). Five studies found no significant change in sleep latency between the conditions (Heath et al., 2014; Grønli et al., 2016; Rångtell et al., 2016; Knufinke et al., 2018; Yang et al., 2018). Summarizing this, three out of eight studies suggested that blue light exposure increased sleep latency.

### 3.3 Blue light and performance

#### 3.3.1 Age, intervention, and duration

Twenty-three studies (see Table 4) were included to investigate the influence of blue light on performance. The participants were aged on average 29.2 years. Nineteen studies compared blue light with a different colored light exposure such as white, red, yellow, amber or green light. Four studies used electronic devices such as smartphones, tablets and computers and compared them with situations where the participants used blue light filters such as blue light blocking glasses or blue light filters for displays or hard-copy books (Heath et al., 2014; Slama et al., 2015; Heo et al., 2017; Driller and Uiga, 2019). Two studies compared blue light exposure to caffeine use (Taillard et al., 2012; Beaven and Ekström, 2013). The average exposure time was 1.7 h. Those studies with longer exposure times were separately calculated and they averaged around 2.5 weeks. The exact exposure times for each study were listed in Table 4.

**TABLE 4:** Influence of blue light on performance.

Study	Age (years) participants (*n*)	Activity	Intervention/Exposure	Duration of intervention	Measurement tool	Methodological characteristics	Outcome
Alkozei et al. (2016)	22.0	Sitting	BL (469 nm) vs. amber light (578 nm)	30 min	N-back task	Control study	Accuracy no significant change in BL
	*n* = 35						Reaction time significant ↓ in BL
An et al. (2009)	20.9 ± 1.1	Oddball task	BL (458 nm) vs. GL (550 nm)	20 min	ERP (P300)	Controlled study	Cognitive function is significant ↑ in BL
	*n* = 12						Reaction time no significant change in BL
Baek and Min (2015)	24.5	Mental task in the early afternoon	BL (451 nm) vs. dark light vs. WL	1 h	CPT, EEG	Crossover study	Reaction times no significant ↓ in BL
	*n* = 20						
Beaven and Ekström (2013)	26 ± 4	Sitting and listening to relaxing music in the late afternoon	BL (40 lux, 470 nm) vs. CAF vs. Placebo (WL and sugar)	1 h	Go/NoGo	RCT	Go/NoGO reaction times significant ↓ in blue-eyed individuals in BL
	*n* = 24						Go/NoGO task-accuracy no significant change in BL
Cajochen et al. (2011)	23.8 ± 5.0	Watching a relaxing movie and performing tasks	BL computer screen, (6.953 K LED, 440–470 nm) vs. CCFL (4.775 K)	5 h	Go/NoGo task, word pair learning task	Controlled crossover study	Go/NoGo performance significant ↑ BL
	*n* = 13						Sustained attention ↑ in BL
Driller and Uiga (2019)	28 ± 5.0	Reading before bedtime	iPad vs. hard copy book	1 h	Heart rate monitor	RCT, crossover study	Resting and exercising heart rates no significant change
	*n* = 14						
Figueiro et al. (2009)	21–46	Sitting still	BL (470 nm) vs. RL (630 nm) vs. preceding dark conditions	April/May	EEG, PVT, self reports of sleepiness	Controlled study	Heart rate significant ↑ in BL and RL
	*n* = 14			45 min			
Gabel et al. (2013)	23.1 ± 0.8	Cognitive tasks during morning hours	BL (470 nm) vs. DSL	20 min	N-back task, PVSAT SART, 5-cognitive tasks	Controlled study	Cognitive performance no significant change in BL
	*n* = 17						
Heath et al. (2014)	17.4 ± 1.9	Playing games before bedtime	iPad vs. iPad with short wavelength filter	48 min	Go/NoGo task	Counterbalanced controlled study	Accuracy (Go/NoGo task) significant ↑ in BL
	*n* = 16						Cognitive alertness no significant change in BL
Heo et al. (2017)	31.0 ± 4.2	Smartphone use during the morning	Smartphone vs. Smartphone display filter	150 min	CPT	RCT	Commission error significant ↑ in BL
	n = 22						
Knaier et al. (2017a)	18–35	Endurance sports	BL (469 nm) vs. bright light vs. control light	60 min	Handgrip strength test	RCT	Handgrip strength and reaction time no significant change in BL
	*n* = 72						
Lehrl et al. (2007)	63.5	Reading and writing	BL (455 nm) vs. YL (580 nm) vs. WL	20 min	Degree of alertness on a 7-step rating scale	Longitudinal study	Alertness significant ↑ in BL
	*n* = 44						
Lockley et al. (2006)	23.3 ± 2.4	Sitting still	BL (460 nm) vs. 555 nm-light	6.5 h	Auditory PVT	RCT	Mean auditory reaction times significant ↓ in BL
	*n* = 16						
Motamedzadeh et al. (2017)	30.2 ± 4.1	Sitting during night shift	BL (17 K) vs. BL (6.5 K) vs. WL	1 week	N-back task	RCT	Cognitive performance (working memory) significant ↑ in BL 17 K
							Omission errors significant ↓ in BL 17 K
	*n* = 30						Reaction time significant ↓ in BL 17 K
							Sustained attention significant ↑ in BL 17 K
Münch et al. (2016)	23.2 ± 3.3	Bedtime routine	BL (750 lux, 3,537 K) vs. OL (100 lux, 1,500 K) vs. CTRL (40 lux, 2,600 K)	30 min	PVT	RCT	PVT reaction times significant ↓ in BL
	*n* = 18						
Phipps-Nelson et al. (2009)	32.1 ± 6.8	Driving during the night	BL (430 nm) vs. RL (620 nm)	6 h	EEG, PVT, STI driving Simulator	RCT	Driving simulator lane deviations; no significant change across the night in all light conditions
	*n* = 8						PVT reaction times significant ↓ in BL
Sahin and Figueiro (2013)	20.8	Sitting, post lunch dip	BL (470 nm) vs. RL (630 nm)	48 min	EEG	RCT	Alertness (alpha and theta waves) no significant change in BL
	*n* = 13						
Scheuermaier et al. (2018)	63.3	Sitting in the evening	BL (320 μW/cm2) vs. WL (370 μW/cm2)	2 h	Actiwatch, DSST, EEG	RCT	Cognitive function (DSST) no significant change in BL
	*n* = 10						
Slama et al. (2015)	22.1 ± 2.2	Sitting and watching a documentary, post lunch	BL (460 nm) and wake group vs. Nap	30 min	PVT	Controlled study	Accuracy no significant ↑ in BL
	23.4 ± 1.6						
	*n* = 25						
Taillard et al. (2012)	33.2 ± 10.9	Driving during the night	BL (468 nm) vs. CAF vs. placebo (decaffeinated coffee)	4 h	ILC during BL	RCT, crossover study	Driving performance significant strong ↑ with CAF and significant moderate ↑ in BL
	*n* = 44						
Tulppo et al. (2014)	25 ± 5	Earbuds light treatment during hockey training	BL vs. Sham	12 min	PST with visual warning signals	RCT	Motor time with a visual warning signal significant ↓ in BL
	*n* = 11						
Viola et al. (2008)	36.4 ± 10.2	Office work during the day	BL (420–480 nm) vs. WL	4 weeks	Likert scale, Questionnaire	Controlled crossover study	Alertness significant ↑ in BL
							Concentration significant ↑ in BL
	*n* = 94						Daytime dysfunction significant ↑ in BL
							Work performance significant ↑ in BL
Yang et al. (2018)	20 ± 3.4	Nightshift work	Intermittent BL (6000 K) vs. continuous bright light vs. continuous dim light (3600 K)	30 min	PVT	RCT	Reaction time significant ↓ in BL
	*n* = 15						Subjective alertness significant ↑ in BL and in continuous bright light

Increase (↑), decrease (↓), blue light (BL), caffeine (CAF), cold cathode fluorescent lamp (CCFL), Continuous Performance Test (CPT), control lighting condition (CTRL), dawn simulation light (DSL), 90-s Digit-Symbol Substitution Test (DSST), electroencephalography (EEG), event related potential (ERP), green light (GL), inappropriate line crossings (ILC), thousand (K), light emitting diode (LED), working memory task (n-back task), Psychomotor Speed Test (PST), Paced Visual Serial Addition Task (PVSAT), Psychomotor Vigilance Task (PVT), randomized controlled trial (RCT), red light (RL), Sustained Attention to Response Task (SART) and Systems Technology Incorporated driving simulator (STI), watt (W), white light (WL) and yellow light (YL).

#### 3.3.2 Activity and measurement

Activities during the blue light exposure included relaxed sitting, bedtime routine, reading and texting on an electronic device, mundane tasks on a smartphone such as Facebook use and playing games, cognitive tasks, oddball tasks, driving a car, office work, and physical exercise. Methods most commonly used to measure the influence of blue light on sleep included: Psychomotor Vigilance Task (PVT), electroencephalography (EEG), working memory task (n-back task) and Go/NoGo task. The following methods of measurement were used less frequently across the included studies: Continuous Performance Test (CPT), 90-s Digit-Symbol Substitution Test (DSST), event related potential P300 (ERP), handgrip strength, inappropriate line crossings (ILC) during a driving task, Likert scale, Paced Visual Serial Addition Task (PVSAT), Psychomotor Speed Test (PST), Actiwatch, Sustained Attention to Response Rask (SART) and Systems Technology Incorporated driving simulator (STI) (Table 4). These methods of measurements were applied either before, during or after the blue light intervention.

#### 3.3.3 Performance responses to blue light

The term performance includes the elements cognitive performance, alertness, reaction times, accuracy, daytime dysfunction, heart rate response, and handgrip strength. More details to each element are given in the following sections.

##### 3.3.3.1 Cognitive performance

Two studies reported an increase in cognitive performance when exposed to the blue light condition (An et al., 2009; Motamedzadeh et al., 2017). In alignment with these finding, one study reported an increase in office work performance when exposed to the blue light condition (Viola et al., 2008) and another showed an increase in driving performance when exposed to the blue light condition (Taillard et al., 2012). However, no significant difference between light conditions was found during simulated driving (Phipps-Nelson et al., 2009). Two studies showed no difference in cognitive performance following blue light exposure on the previous evening (Gabel et al., 2013; Scheuermaier et al., 2018). Summarizing these findings as “cognitive performance,” four out of seven studies reported blue light to increase cognitive performance.

##### 3.3.3.2 Alertness

Three studies reported of increase in alertness when exposed to the blue light condition (Lehrl et al., 2007; Viola et al., 2008; Yang et al., 2018). Two studies reported an increase in sustained attention when exposed to the blue light condition (Cajochen et al., 2011; Motamedzadeh et al., 2017). One study reported an increase in concentration in the blue light condition (Viola et al., 2008). One study reported a decrease in omission errors (Motamedzadeh et al., 2017) after blue light exposure. Two studies found no significant change in cognitive alertness when exposed to the blue light condition (Sahin and Figueiro, 2013; Heath et al., 2014). One study reported of making more commission errors in a CPT the next morning after exposure to the blue light condition (Heo et al., 2017). In summary, there were seven occurrences out of ten occurrences, counting the references Motamedzadeh et al. (2017), Viola et al. (2008) twice for multiple results, which reported blue light to be increasing alertness.

##### 3.3.3.3 Reaction times and accuracy

Seven studies reported a decrease in reaction times when exposed to the blue light condition (Lockley et al., 2006; Phipps-Nelson et al., 2009; Tulppo et al., 2014; Alkozei et al., 2016; Münch et al., 2016; Motamedzadeh et al., 2017; Yang et al., 2018). One study reported a decrease in reaction times in blue-eyed participants during blue light exposure (Beaven and Ekström, 2013). Additionally, one study reported improved performance in the Go/NoGo task when exposed to the blue light condition (Cajochen et al., 2011). Four studies showed no significant change in reaction times when exposed to the blue light condition (An et al., 2009; Tulppo et al., 2014; Baek and Min, 2015; Knaier et al., 2017a). In summary, nine out of thirteen studies showed blue light to decrease reaction time. Blue light exposure only increased accuracy in one study (Heath et al., 2014), whilst three studies found no significant change in accuracy (Beaven and Ekström, 2013; Slama et al., 2015; Alkozei et al., 2016).

##### 3.3.3.4 Daytime dysfunction

One study reported daytime dysfunction to be increased when exposed to the blue light condition (Viola et al., 2008).

##### 3.3.3.5 Heart rate and handgrip strength

One study found that heart rate was increased when exposed to the blue light condition, but also the red light condition compared to the dark condition (Figueiro et al., 2009). Another study found no significant effect on heart rate the next morning after evening exposure to the blue light (Driller and Uiga, 2019). Handgrip strength was not influenced by exposure to the blue light condition (Knaier et al., 2017a).

### 3.4 Blue light and wellbeing

#### 3.4.1 Age, intervention, and duration

Eight studies were included to investigate the influence of blue light on wellbeing (Table 5). The participants were aged on average 29.5 years, one study did not mention the exact age of their participants but stated that they were all adults (Rångtell et al., 2016). Four studies compared blue light with a different colored light such as white, red, green or orange light (Table 5). Four studies used electronic devices such as smartphones, tablets and computers and compared them with situations where the participants used blue light filters such as blue light blocking glasses or blue light filters for displays (Table 5). Additionally, one study compared blue light exposure to caffeine use (Beaven and Ekström, 2013). The average exposure time was 1.6 h. Those studies with longer exposure times were separately calculated and they averaged around 3.5 weeks. The exact exposure times for each study were listed on Table 5.

**TABLE 5:** Influence of blue light on wellbeing.

Study	Age (years) participants (*n*)	Activity	Intervention/Exposure	Duration of intervention	Measurement tool	Methodological characteristics	Outcome
Burkhart and Phelps (2009)	34 ± 8.2	Bedtime routine	Yellow tinted safety glasses vs. BLB amber glasses	3 h	PANAS	Controlled study	Mood significant ↑ with BLB glasses
	*n* = 20						
Driller and Uiga (2019)	28 ± 5.0	Reading before bedtime	iPad vs. hard copy book	1 h	Likert scale	RCT, crossover study	Motivation to exercise no significant change in BL perceived during exercise and no significant change on the following day
	*n* = 14						
Ekström and Beaven (2014)	26 ± 4	Sitting during the morning	BL (40 lux, 470 nm) vs. CAF vs. Placebo (WL and sugar)	1 h	SCAS	RCT, crossover study	Mood significant strong ↑ in (BLxCAF) compared to mood significant moderate ↑ in (BLxplacebo)
	*n* = 20						
Gabel et al. (2013)	23.1 ± 0.8	Cognitive tasks during morning hours	BL (470 nm) vs. DSL	20 min	PANAS, VAS	Controlled study	Subjective wellbeing no significant change in BL
	*n* = 17						
Heo et al. (2017)	31.0 ± 4.2	Smartphone use during the morning	Smartphone vs. Smartphone display filter	150 min	POMS	RCT, crossover study	Tension and anxiety no significant change in BL
	*n* = 22						
Iskra-Golec et al. (2012)	28.3 ± 2.8	Sitting	BL (17 K, 420 nm–480 nm) vs. WL (4 K)	3 weeks, office hours	Polish adaptation of the UWIST mood adjective check list	Field experiment counterbalanced	Energetic arousal significant ↑ in BL during the morning
	*n* = 30						Hedonic tone and tense arousal no significant change in BL
Rångtell et al. (2016)	adults	Reading a book	Tablet vs. hard copy	2 h	KSS	RCT	Subjective ratings of arousal and other feelings no significant change in BL
	*n* = 14						
Viola et al. (2008)	36.4 ± 10.2	Office work during the day	BL (420–480 nm) vs. WL	4 weeks	H&ES, KSS, PANAS	Controlled crossover study	Irritability significant ↓ in BL
	*n* = 94						Positive mood significant ↑ in BL

Increase (↑), decrease (↓), blue light (BL), blue light blocking (BLB), caffeine (CAF), dawn simulation light (DSL), Headache and Eye Strain scale (H&ES), thousand (K), Karolinska Sleepiness Scale (KSS), Positive And Negative Affect Schedule (PANAS), Profile Of Mood States (POMS), randomized control trials (RCT), Swedish Core Affect Scales (SCAS), University of Wales Institute of Science and Technology (UWIST), Visual Analogue Scale (VAS), and white light (WL).

#### 3.4.2 Activity and measurement

Activity during the blue light exposure included relaxed sitting, leisure, bedtime routine, reading on an electronic device, mundane tasks on a smartphone such as Facebook use and playing games, cognitive tasks, and office work. Methods to measure the influence of blue light on sleep were: Positive And Negative Affect Schedule (PANAS), Visual Analogue Scale (VAS) and Karolinska Sleepiness Scale (KSS). The following methods of measurement were used less frequently across the included studies: Headache and Eye Strain scale (H&ES), Likert scale, Profile of Mood States (POMS) and Polish adaptation of the University of Wales Institute of Science and Technology (UWIST) mood adjective check list (Table 5). Those methods were used to measure the influence of blue light either before, during or after the intervention.

#### 3.4.3 Wellbeing responses to blue light

The term wellbeing includes the elements mood, irritability, arousal, tension, anxiety, and motivation. More details to each element are given in the following sections.

##### 3.4.3.1 Mood

Two studies found an increase in positive mood when exposed to blue light (Viola et al., 2008; Ekström and Beaven, 2014). One study showed higher results for mood in the non-blue light condition, adding to the evidence that blue light can decrease mood (Burkhart and Phelps, 2009). However, one study showed no change in subjective wellbeing in the blue light condition (Gabel et al., 2013).

##### 3.4.3.2 Irritability

One study reported irritability to be decreased when exposed to the blue light condition (Viola et al., 2008).

##### 3.4.3.3 Arousal

One study showed an increase in energetic arousal in the blue light condition (Iskra-Golec et al., 2012). For tense arousal, hedonic tone, no significant changes were found (Iskra-Golec et al., 2012). Likewise no significant change was found for subjective arousal and other feelings (Rångtell et al., 2016).

##### 3.4.3.4 Tension and anxiety

One study reported tension and anxiety following the blue light condition not to be significantly affected (Heo et al., 2017).

##### 3.4.3.5 Motivation

The motivation to exercise and the perceived exertion during exercise on the following day was reported not to be influenced by blue light exposure (Driller and Uiga, 2019).

## 4 Discussion

This systematic review summarized the current data of blue light exposure and its influence on sleep, performance and wellbeing. One half of the study results found tiredness and sleep efficacy to be decreased by blue light exposure. Sleep quality, sleep duration, and sleep latency did not seem to be systematically affected by blue light exposure. Most studies found cognitive performance and alertness to be increased and reaction time decreased by blue light exposure. The wellbeing markers mood, irritability, arousal, tension, and anxiety were shown to be increased by blue light exposure by slightly less than half of the included studies.

### 4.1 Sleep

It is the norm to measure restful and good sleep based on sleep quality. However, sleep can also be perceived as restful if the next day’s tiredness was low, sleep efficacy was high or sleep latency short. Sleep is a very wide-ranging term. In addition to sleep quality, other elements of sleep, such as tiredness, sleep duration, sleep efficacy and sleep latency can help to interprete sleep health. Recently, a growing number of studies research the influence of sleep on athletic performance. One reason for this might be the belief that a good night’s sleep is the foundation for good performance (Samuels et al., 2016). Thus, it is of interest to athletes to build an evidence base investigating the influence of blue light exposure on sleep, especially as the use of electronic devices has become a permanent feature of our everyday life.

#### 4.1.1 Tiredness

Tiredness due to sleep deprivation or physical strain might decrease performance. Since tiredness is a broad term summarizing sleepiness, fatigue, wakefulness, arousal, and morning alertness as one, other terms, which are related to the terms listed, can be used to draw parallels to the term tiredness. For example, mental fatigue is not explicitly named among these terms, but its meaning is similar to the other terms describing tiredness. It was found that mental fatigue has an influence on physical performance as well as on cognitive performance (Van Cutsem et al., 2017). This means that if mental fatigue influences physical performance, tiredness as a whole might also influence physical performance. Thus, tiredness might be a relevant factor for athletes to consider if they want to improve their performance. The included studies showed that out of the 20 occurrences reporting tiredness to be influenced by blue light, 10 occurrences found a positive effect of blue light exposure as it decreases tiredness (Table 3). The significance of this finding is that athletes might be able to use blue light exposure to reduce their tiredness before a competition. In addition, given the connection between physical performance and tiredness (Van Cutsem et al., 2017), blue light exposure may indeed improve an athlete’s physical performance, reduce the risk of injury and help in staying focused. Regarding the influence of tiredness on decision making, there is one study that found decision making not to be influenced by fatigue and therefore not by tiredness (Almonroeder et al., 2020). In case an athlete shows signs of tiredness on competition days, about 2 h of blue light exposure might help to reduce tiredness, which was the average time of the included studies (Table 3). A more practical choice of blue light exposure might be the use of smartphones. However, this does not mean that electronic devices are better than blue light bulbs because this systematic review cannot show relevant differences in the results regarding the two intervention methods. Since almost exclusively, questionnaires were used, and rating tiredness is often subjective (Enoka and Duchateau, 2016), this raises the question of reliability of these results. Among the questionnaires, the KSS was most often used, which appears to be a valid tool to assess tiredness as it correlates with EEG measurements (Kaida et al., 2006). For further research using questionnaires with unknown reliability and validity, adding a PVT, which measures reaction time, might increase the objectivity of test results. The assumption here is that when tiredness is increased, performance consequentially will be decreased (Brown et al., 2013) and the result of the PVT will worsen. Additionally, athletic self-report tests based on parameters such as heart-rate or jump test data could help to quantify tiredness as stated (Thorpe et al., 2017). Further research should explore the interaction of blue light and tiredness with the focus on the relationship between tiredness and risk of injury, staying focused and motivated.

#### 4.1.2 Sleep quality

Even though blue light can have a positive effect by reducing tiredness, the evidence is mixed. This systematic review found that three out of five studies showed no significant change of sleep quality by blue light exposure (Table 3), whilst two studies found sleep quality to be influenced by blue light. One study found blue light to be increasing sleep quality, the other found it to be decreasing. However, it is clear that the consequence of reduced sleep quality is bad sleep. Additionally it was recently found that bad sleep decreases performance and recovery (Hamlin et al., 2021). Both are very important factors for athletes, because performance and recovery are the foundation of their success. Caution is advised when making suggestion to use blue light exposure to improve sleep quality, as it may actually negatively affect an athlete’s sleep and recovery. Since Burkhart and Phelps (2009) found a decrease in sleep quality after 3 h of blue light exposure, it might be recommended to restrict the usage of blue light emitting devices 3 h before bedtime. With that, sleep quality should not be decreased and the athlete might rest better. Additionally, it was found that an improvement in sleep quality can cause an increase in reaction time, accuracy, endurance performance and a decrease in injury and illness (Krystal and Edinger, 2008). Restricting the usage of blue light emitting devices before bedtime might be a harsh interference with the daily habits of the athlete. Therefore, coach and athlete should talk about the advantages of a sensible usage of blue light and decide together how they want to restrict the blue light exposure in the athlete’s daily life. It might be enough to only restrict the use of blue light emitting devices some hours before bedtime during periods of great physical exertion, for example during intense training camps or before competitions. Sleep quality might have an influence on tiredness and how fatigue is perceived (Lavidor et al., 2003). It was found that fatigue was appearing alongside bad sleep quality (Fortier-Brochu et al., 2010). Combining these two findings, this might mean that reduced tiredness after a night’s rest, is a sign of better sleep quality. Summarized, these findings might suggest that by improving sleep quality, tiredness is reduced on a subjective level and physical performance might improve. Further research should focus on the influence of blue light exposure on sleep quality during intense training programs and competition, because those are the times when good performance and good recovery, which might be dependent on good sleep quality, matter the most. As for the reliability of the studies included it was found that exclusively questionnaires were used. Whilst the PSQI used in some studies is reported of having a high reliability and a good validity (Backhaus et al., 2002). Future studies should evaluate if more objective forms of measurements such as PSG, NREM sleep EEG, and actigraphy (Backhaus et al., 2002), are needed and worth the time to increase objectivity and reliability.

#### 4.1.3 Sleep duration, sleep efficacy, sleep latency

Three out of nine studies found sleep duration to be decreased by blue light exposure and one study found it to be increased (Table 3). There are arguments for and against sleep duration being relevant for athletes. On one hand athletes can suffer from sleep deprivation due to harsh training schedules (Romyn et al., 2016) and if blue light would also increase the needed duration of sleep, then sleep deprivation might only get worse if blue light exposure was not moderated. On the other hand, an argument can be made against sleep duration being relevant because compared to sleep quality, sleep duration might have less influence on perceived fatigue (Fortier-Brochu et al., 2010). This might mean that sleep duration is less relevant than sleep quality when assessing an athlete’s sleeping habits. Other quantitative measurements of sleep are sleep efficacy and sleep latency. With two showing sleep efficacy to be increased by blue light exposure, two others found no effect (Table 3). These results are similar to sleep duration as it describes the ratio of sleep time to bedtime (Reed and Sacco, 2016). Sleep latency can be interpreted alongside sleep efficacy as it describes the time spent in bed until the participant falls asleep. Since sleep latency is related to sleep efficacy and sleep duration, similar interpretations and consequences are expected. Three out of eight studies found an increase in sleep latency through blue light exposure (Table 3). Further research is needed to evaluate if sleep duration, sleep efficacy, and sleep latency are influenced by blue light exposure.

### 4.2 Performance

Sleep, caffeine and even small rituals, which put the athlete in the right headspace can improve performance (Broch and Kristiansen, 2014; Spriet, 2014). But blue light is not something that is usually considered when preparing for a competition. Blue light is not expected to directly improve the physical aspect of performance, but rather the mental aspects such as recognizing opportunities, planning and decision-making. However, further studies will be needed to assess the exact influence of blue light exposure on these mental aspects.

#### 4.2.1 Cognitive performance and alertness

Cognitive performance describes the abilities of paying attention, memorizing, decision-making, planning, and reasoning (Dhakal and Bobrin, 2022). Improving cognitive performance with blue light exposure might be useful for sports which include teamwork, decision-making and quickly changing situations. Additional effects of improving cognitive performance include the prevention of injury because the athlete might be more aware of their surroundings. With four out of seven studies, the slight majority found cognitive performance to be increased by blue light exposure (Table 4). The validity and reliability of the measurement of cognitive performance is not clear. EEG, n-back tasks, driving, H&ES and PVT were used, but there were no studies which investigated the validity and reliability of these tests for cognitive performance. For further research other valid methods should be explored to measure cognitive performance. For example, the Stroop Color and Word Test (SCWT) is a color/word test measuring cognitive function (Stroop, 1935) and the Test of Attentional Performance (TAP) is a test including 13 different subtests, like Go/Nogo tests, working memory and alertness tests (Zimmermann and Fimm, 2002). Since the definition of alertness and attention are close, and the latter is part of cognitive performance, alertness might also be interpreted alongside cognitive performance. With seven out of ten occurrences, a slight majority regarding alertness showed an increased alertness through blue light exposure (Table 4). Since cognitive performance was also increased, the increase for alertness is what was expected. The studies used reliable methods such as CPT (Raz et al., 2014) and n-back task (Jaeggi et al., 2010) to assess alertness. PVT is also a good method, but research has shown that there is need to calibrate PVT in specific margins to give reliable answers (Basner et al., 2021). The Likert scale was described as invalid and not reliable (Louangrath, 2018), so the results achieved for two mentioned studies with it (Lehrl et al., 2007; Viola et al., 2008), should be carefully assessed.

#### 4.2.2 Reaction times and accuracy

Sports which include teamwork, decision-making and quick changing situations often put athletes in situations where performance is determined by who recognizes the situation faster and is quicker to act accordingly. This process is what the term reaction time describes, which is influenced by recognizing the situation, decision-making and taking action. With nine out of thirteen studies, the majority found reaction time to be improved by blue light exposure (Table 4). Since assessing situations and planning are part of cognitive performance, this raises the question on how reaction time and cognitive performance are related. Reaction time has mental and motor aspects, whereas cognitive performance has only mental aspects. Since both cognitive performance and reaction time are influenced by blue light, this might suggest that reaction time and cognitive performance are connected. There might be evidence for that to be the case, since reaction time is measured with PVT and PVT is used in multiple studies to measure cognitive performance. As for the measurement of CPT, n-back tasks and PVTs were used by the included studies and were described as reliable methods (Jaeggi et al., 2010; Raz et al., 2014; Basner et al., 2021). Further research should test these findings in an athletic setting to find out if blue light exposure can make a difference in an athlete’s mental performance. Research should further investigate howblue light influences accuracy since there were only a total of four studies addressing this parameter, out of which one study (Heath et al., 2014) showed accuracy to be increased by blue light exposure.

#### 4.2.3 Driving

Accident-free driving is an important health and safety issue, which might potentially be influenced by blue light exposure. In fact, two studies (Phipps-Nelson et al., 2009; Taillard et al., 2012) investigated this topic. Whereas one study showed a moderate but significant increase in driving performance (Taillard et al., 2012) the other did not find any difference between the blue light and the red light condition (Phipps-Nelson et al., 2009), although the blue light exposure significantly decreased PVT reaction time. However, further studies are warranted to elucidate this important health and safety issue.

### 4.3 Wellbeing

The wellbeing of athletes is strained because they are under a lot of pressure to perform well. The desire to perform well might lead to overtraining and mental stress. High stress levels might distract athletes from their optimal performance in competitions. A study stated that stress might lead to injury and issues with their mental health (Rice et al., 2016). Improving wellbeing might hence allow athletes to improve their physical performance. Interestingly, blue light exposure may help relieve athletes of stress and confer relaxation. This systematic review summarized mood, irritability, arousal, tension, and anxiety under the term wellbeing: five out of ten occurrences found blue light exposure to be influencing wellbeing. Out of these five occurrences four found wellbeing to be increased by blue light exposure (Table 5). This suggests that blue light may help athletes to improve their performance. However, there is need for more research regarding the influence of wellbeing on performance. A connection between performance and wellbeing was observed but its underlying mechanism is not understood (Daniels and Harris, 2000). Interestingly, there is evidence for a correlation between injury and stress (Lavallée and Flint, 1996). This might mean, that if an improvement in wellbeing equals lower stress levels, frequency of injury might be decreased. Further research could investigate the influence of blue light on stress levels and survey the occurrence of injury and illness. The review showed that electronic devices and light bulbs emission can improve wellbeing. Athletes could use their smartphones immediately before competitions to distract them from stress and use the blue light from the smartphone as an additional effect to improving wellbeing. As for the question if the research methodology is valid: PANAS, UWIST, and VAS are all valid and reliable methods to measure wellbeing (Matthews et al., 1990; Kreindler et al., 2003; Crawford and Henry, 2004), and POMS was described as a consistent form of measurement (Norcross et al., 1984).

### 4.4 Limitations

The participants were healthy humans and on average between 20 and 30 years old. The discussed results were applied to athletes, because the average age of the participants lies in the expected age range of athletes. Only three studies (Tulppo et al., 2014; Knaier et al., 2017a; Knufinke et al., 2018) included athletes as study participants. The included studies focused on slightly different research questions, for this reason multiple terms, e.g., sleepiness and fatigue, were summarized under one umbrella term, e.g., tiredness. Since participants were exposed to blue light during mostly non-strenuous and non-athletic tasks, the influence of physical stress and hormones was left unexplored. The influence of mental health on wellbeing and performance and any influence of blue light was not assessed. There is evidence for interactions between sleep, performance and wellbeing, but exploring these further goes beyond the scope of the discussion. To be able to give definitive answers to the question if blue light influences athletes and athletic performance, further studies are needed which specifically discuss the influence of blue light on athletes in different situations. Further, the question if blue light exposure during sleep time (e.g., checking the smart phone at 2 a.m. in the morning) or during actual sleep might be harmful remains unanswered by our review. Thus, further investigations seem warranted so clarify this important topic.

## 5 Conclusion

This systematic review has shown that blue light exposure might influence sleep, performance and wellbeing. A majority of studies found positive effects such as an increase in cognitive performance, alertness and reduced reaction times. Improving cognitive performance is useful in sports which require team-work and decision-making. Additionally, injury might be prevented by increased levels of alertness. Increased wellbeing might reduce stress and therefore lessen the risk of injury. An important negative effect of blue light exposure might be a decrease in sleep quality and sleep duration, because it might negatively influence performance and recovery. However, in general, the specific effects of blue light exposure seem still to be a murky field and more investigations are needed before final firm and evidence-based conclusions can be drawn. Based on our present findings, further research is recommended to determine if blue light exposure can improve athletic performance in specific aspects by influencing sleep, performance and wellbeing.

## Data Availability

The original contributions presented in the study are included in the article/supplementary material, further inquiries can be directed to the corresponding author.

## References

[B1] Alkozei A. Smith R. Pisner D. A. Vanuk J. R. Berryhill S. M. Fridman A. (2016). Exposure to blue light increases subsequent functional activation of the prefrontal cortex during performance of a working memory task. Sleep 39, 1671–1680. 10.5665/sleep.6090 27253770 PMC4989256

[B2] Almonroeder T. G. Tighe S. M. Miller T. M. Lanning C. R. (2020). The influence of fatigue on decision-making in athletes: A systematic review. Sports Biomech. 19, 76–89. 10.1080/14763141.2018.1472798 29902127

[B3] An M. Huang J. Shimomura Y. Katsuura T. (2009). Time-of-day-dependent effects of monochromatic light exposure on human cognitive function. J. Physiol. Anthropol. 28, 217–223. 10.2114/jpa2.28.217 19823003

[B4] Ayaki M. Hattori A. Maruyama Y. Nakano M. Yoshimura M. Kitazawa M. (2016). Protective effect of blue light shield eyewear for adults against light pollution from self-luminous devices used at night. Chronobiol. Int. 33, 134–139. 10.3109/07420528.2015.1119158 26730983

[B5] Backhaus J. Junghanns K. Broocks A. Riemann D. Hohagen F. (2002). Test-retest reliability and validity of the Pittsburgh sleep quality Index in primary insomnia. J. Psychosom. Res. 53, 737–740. 10.1016/s0022-3999(02)00330-6 12217446

[B6] Baek H. Min B.-K. (2015). Blue light aids in coping with the post-lunch dip: An EEG study. Ergonomics 58, 803–810. 10.1080/00140139.2014.983300 25559376

[B7] Basner M. Moore T. M. Nasrini J. Gur R. C. Dinges D. F. (2021). Response speed measurements on the Psychomotor Vigilance Test: How precise is precise enough? Sleep 44, zsaa121. 10.1093/sleep/zsaa121 32556295 PMC8240655

[B8] Beaven C. M. Ekström J. (2013). A comparison of blue light and caffeine effects on cognitive function and alertness in humans. PLoS ONE 8, e76707. 10.1371/journal.pone.0076707 24282477 PMC3838207

[B9] Bowler J. Bourke P. (20191953). Facebook use and sleep quality: Light interacts with socially induced alertness. Br. J. Psychol. 110, 519–529. 10.1111/bjop.12351 PMC6767460 30291634

[B10] Broch T. B. Kristiansen E. (2014). The margin for error”: Ritual coping with cultural pressures. Scand. J. Med. Sci. Sports 24, 837–845. 10.1111/sms.12077 23662772

[B11] Brown T. Johnson R. Milavetz G. (2013). Identifying periods of drowsy driving using EEG. Ann. Adv. Automot. Med. 57, 99–108. 24406950 PMC3861841

[B12] Burkhart K. Phelps J. (2009). Amber lenses to block blue light and improve sleep: A randomized trial. Chronobiol. Int. 26, 1602–1612. 10.3109/07420520903523719 20030543

[B13] Cajochen C. Frey S. Anders D. Späti J. Bues M. Pross A. (20111985). Evening exposure to a light emitting diodes (LED)-backlit computer screen affects circadian physiology and cognitive performance. J. Appl. Physiol. 110, 1432–1438. 10.1152/japplphysiol.00165.2011 21415172

[B14] Chang A.-M. Aeschbach D. Duffy J. F. Czeisler C. A. (2015). Evening use of light emitting eReaders negatively affects sleep, circadian timing, and next-morning alertness. Proc. Natl. Acad. Sci. U. S. A. 112, 1232–1237. 10.1073/pnas.1418490112 25535358 PMC4313820

[B15] Chellappa S. L. Steiner R. Oelhafen P. Lang D. Götz T. Krebs J. (2013). Acute exposure to evening blue-enriched light impacts on human sleep. J. Sleep. Res. 22, 573–580. 10.1111/jsr.12050 23509952

[B16] Chindamo S. Buja A. DeBattisti E. Terraneo A. Marini E. Gomez Perez L. J. (2019). Sleep and new media usage in toddlers. Eur. J. Pediatr. 178, 483–490. 10.1007/s00431-019-03318-7 30652219

[B17] Crawford J. R. Henry J. D. (2004). The Positive and Negative Affect Schedule (PANAS): Construct validity, measurement properties and normative data in a large non-clinical sample. Br. J. Clin. Psychol. 43, 245–265. 10.1348/0144665031752934 15333231

[B18] Daniels K. Harris C. (2000). Work, psychological wellbeing and performance. Occup. Med. 50, 304–309. 10.1093/occmed/50.5.304 10975125

[B19] Dhakal A. Bobrin B. D. (2022). “Cognitive deficits,” in StatPearls (Treasure Island (FL): StatPearls Publishing). Available at: http://www.ncbi.nlm.nih.gov/books/NBK559052/(Accessed March 10, 2022). 32644478

[B20] Driller M. Uiga L. (2019). The influence of night-time electronic device use on subsequent sleep and propensity to be physically active the following day. Chronobiol. Int. 36, 717–724. 10.1080/07420528.2019.1588287 30889985

[B21] Ekström J. G. Beaven C. M. (2014). Effects of blue light and caffeine on mood. Psychopharmacol. (Berl.) 231, 3677–3683. 10.1007/s00213-014-3503-8 24590053

[B22] Enoka R. M. Duchateau J. (2016). Translating fatigue to human performance. Med. Sci. Sports Exerc. 48, 2228–2238. 10.1249/MSS.0000000000000929 27015386 PMC5035715

[B23] Figueiro M. G. Bierman A. Plitnick B. Rea M. S. (2009). Preliminary evidence that both blue and red light can induce alertness at night. BMC Neurosci. 10, 105. 10.1186/1471-2202-10-105 19712442 PMC2744917

[B24] Fortier-Brochu E. Beaulieu-Bonneau S. Ivers H. Morin C. M. (2010). Relations between sleep, fatigue, and health-related quality of life in individuals with insomnia. J. Psychosom. Res. 69, 475–483. 10.1016/j.jpsychores.2010.05.005 20955867 PMC2958173

[B25] Gabel V. Maire M. Reichert C. F. Chellappa S. L. Schmidt C. Hommes V. (2013). Effects of artificial dawn and morning blue light on daytime cognitive performance, wellbeing, cortisol and melatonin levels. Chronobiol. Int. 30, 988–997. 10.3109/07420528.2013.793196 23841684

[B26] Grønli J. Byrkjedal I. K. Bjorvatn B. Nødtvedt Ø. Hamre B. Pallesen S. (2016). Reading from an iPad or from a book in bed: The impact on human sleep. A randomized controlled crossover trial. Sleep. Med. 21, 86–92. 10.1016/j.sleep.2016.02.006 27448477

[B27] Hamlin M. J. Deuchrass R. W. Olsen P. D. Choukri M. A. Marshall H. C. Lizamore C. A. (2021). The effect of sleep quality and quantity on athlete’s health and perceived training quality. Front. Sports Act. Living 3, 705650. 10.3389/fspor.2021.705650 34568820 PMC8461238

[B28] Heath M. Sutherland C. Bartel K. Gradisar M. Williamson P. Lovato N. (2014). Does one hour of bright or short-wavelength filtered tablet screenlight have a meaningful effect on adolescents’ pre-bedtime alertness, sleep, and daytime functioning? Chronobiol. Int. 31, 496–505. 10.3109/07420528.2013.872121 24397302

[B29] Heo J.-Y. Kim K. Fava M. Mischoulon D. Papakostas G. I. Kim M.-J. (2017). Effects of smartphone use with and without blue light at night in healthy adults: A randomized, double-blind, crossover, placebo-controlled comparison. J. Psychiatr. Res. 87, 61–70. 10.1016/j.jpsychires.2016.12.010 28017916

[B30] Hysing M. Pallesen S. Stormark K. M. Jakobsen R. Lundervold A. J. Sivertsen B. (2015). Sleep and use of electronic devices in adolescence: Results from a large population-based study. BMJ Open 5, e006748. 10.1136/bmjopen-2014-006748 PMC4316480 25643702

[B31] Iskra-Golec I. Wazna A. Smith L. (2012). Effects of blue-enriched light on the daily course of mood, sleepiness and light perception: A field experiment. Light. Res. Technol. 44, 506–513. 10.1177/1477153512447528

[B32] Jaeggi S. M. Buschkuehl M. Perrig W. J. Meier B. (2010). The concurrent validity of the N-back task as a working memory measure. Memory 18, 394–412. 10.1080/09658211003702171 20408039

[B33] Kaida K. Takahashi M. Akerstedt T. Nakata A. Otsuka Y. Haratani T. (2006). Validation of the Karolinska sleepiness scale against performance and EEG variables. Clin. Neurophysiol. 117, 1574–1581. 10.1016/j.clinph.2006.03.011 16679057

[B34] Kmet L. M. Lee R. C. Cook L. S. (2004). Standard quality assessment criteria for evaluating primary research papers from a variety of fields. Edmonton: Alberta Heritage Foundation for Medical Research. Alberta Heritage Foundation for Medical Research, A., Health Technology Assessment Unit, U. of C., and Faculty of Medicine, C. H. R.

[B35] Knaier R. Schäfer J. Rossmeissl A. Klenk C. Hanssen H. Höchsmann C. (2017a). Effects of bright and blue light on acoustic reaction time and maximum handgrip strength in male athletes: A randomized controlled trial. Eur. J. Appl. Physiol. 117, 1689–1696. 10.1007/s00421-017-3659-0 28597081

[B36] Knaier R. Schäfer J. Rossmeissl A. Klenk C. Hanssen H. Höchsmann C. (2017b). Prime time light exposures do not seem to improve maximal physical performance in male elite athletes, but enhance end-spurt performance. Front. Physiol. 8, 264. 10.3389/fphys.2017.00264 28507521 PMC5410597

[B37] Knufinke M. Nieuwenhuys A. Geurts S. A. E. Coenen A. M. L. Kompier M. A. J. (2018). Self-reported sleep quantity, quality and sleep hygiene in elite athletes. J. Sleep. Res. 27, 78–85. 10.1111/jsr.12509 28271579

[B38] Kreindler D. Levitt A. Woolridge N. Lumsden C. J. (2003). Portable mood mapping: The validity and reliability of analog scale displays for mood assessment via hand-held computer. Psychiatry Res. 120, 165–177. 10.1016/s0165-1781(03)00196-3 14527648

[B39] Krystal A. D. Edinger J. D. (2008). Measuring sleep quality. Sleep. Med. 9 (Suppl. 1), S10–S17. 10.1016/S1389-9457(08)70011-X 18929313

[B40] Lai Y. Yew Y. (2016). Neonatal blue light phototherapy and melanocytic nevus count in children: A systematic review and meta-analysis of observational studies. Pediatr. Dermatol. 33, 62–68. 10.1111/pde.12730 26645992

[B41] Lastella M. Lovell G. P. Sargent C. (2014). Athletes’ precompetitive sleep behaviour and its relationship with subsequent precompetitive mood and performance. Eur. J. Sport Sci. 14 (Suppl. 1), S123–S130. 10.1080/17461391.2012.660505 24444196

[B42] Lavallée L. Flint F. (1996). The relationship of stress, competitive anxiety, mood state, and social support to athletic injury. J. Athl. Train. 31, 296–299. 16558413 PMC1318911

[B43] Lavidor M. Weller A. Babkoff H. (2003). How sleep is related to fatigue. Br. J. Health Psychol. 8, 95–105. 10.1348/135910703762879237 12643819

[B44] Lawrenson J. G. Hull C. C. Downie L. E. (2017). The effect of blue light blocking spectacle lenses on visual performance, macular health and the sleep-wake cycle: A systematic review of the literature. Ophthalmic Physiol. Opt. 37, 644–654. 10.1111/opo.12406 29044670

[B45] Lehrl S. Gerstmeyer K. Jacob J. H. Frieling H. Henkel A. W. Meyrer R. (2007). Blue light improves cognitive performance. J. Neural Transm. 114, 457–460. 10.1007/s00702-006-0621-4 17245536

[B46] Lockley S. W. Evans E. E. Scheer F. A. J. L. Brainard G. C. Czeisler C. A. Aeschbach D. (2006). Short-wavelength sensitivity for the direct effects of light on alertness, vigilance, and the waking electroencephalogram in humans. Sleep 29, 161–168. 16494083

[B47] Louangrath P. (2018). Reliability and validity of survey scales. 10.5281/zenodo.1322695

[B48] Matthews G. Jones D. M. Chamberlain A. G. (1990). Refining the measurement of mood: The UWIST mood adjective checklist. Br. J. Psychol. 81, 17–42. 10.1111/j.2044-8295.1990.tb02343.x

[B49] Moher D. Shamseer L. Clarke M. Ghersi D. Liberati A. Petticrew M. (2015). Preferred reporting items for systematic review and meta-analysis protocols (PRISMA-P) 2015 statement. Syst. Rev. 4, 1. 10.1186/2046-4053-4-1 25554246 PMC4320440

[B50] Motamedzadeh M. Golmohammadi R. Kazemi R. Heidarimoghadam R. (2017). The effect of blue-enriched white light on cognitive performances and sleepiness of night-shift workers: A field study. Physiol. Behav. 177, 208–214. 10.1016/j.physbeh.2017.05.008 28495465

[B51] Münch M. Nowozin C. Regente J. Bes F. De Zeeuw J. Hädel S. (2016). Blue-enriched morning light as a countermeasure to light at the wrong time: Effects on cognition, sleepiness, sleep, and circadian phase. Neuropsychobiology 74, 207–218. 10.1159/000477093 28637029

[B52] Norcross J. C. Guadagnoli E. Prochaska J. O. (1984). Factor structure of the profile of mood States (POMS): Two partial replications. J. Clin. Psychol. 40, 1270–1277. 10.1002/1097-4679(198409)40:5<1270::aid-jclp2270400526>3.0.co;2-7 6490926

[B53] Phipps-Nelson J. Redman J. R. Schlangen L. J. M. Rajaratnam S. M. W. (2009). Blue light exposure reduces objective measures of sleepiness during prolonged nighttime performance testing. Chronobiol. Int. 26, 891–912. 10.1080/07420520903044364 19637049

[B54] Rångtell F. H. Ekstrand E. Rapp L. Lagermalm A. Liethof L. Búcaro M. O. (2016). Two hours of evening reading on a self-luminous tablet vs. reading a physical book does not alter sleep after daytime bright light exposure. Sleep. Med. 23, 111–118. 10.1016/j.sleep.2016.06.016 27539026

[B55] Raz S. Bar-Haim Y. Sadeh A. Dan O. (2014). Reliability and validity of the online continuous performance test among young adults. Assessment 21, 108–118. 10.1177/1073191112443409 22517923

[B56] Reed D. L. Sacco W. P. (2016). Measuring sleep efficiency: What should the denominator be? J. Clin. Sleep. Med. 12, 263–266. 10.5664/jcsm.5498 26194727 PMC4751425

[B57] Rice S. M. Purcell R. De Silva S. Mawren D. McGorry P. D. Parker A. G. (2016). The mental health of elite athletes: A narrative systematic review. Sports Med. 46, 1333–1353. 10.1007/s40279-016-0492-2 26896951 PMC4996886

[B58] Romyn G. Robey E. Dimmock J. A. Halson S. L. Peeling P. (2016). Sleep, anxiety and electronic device use by athletes in the training and competition environments. Eur. J. Sport Sci. 16, 301–308. 10.1080/17461391.2015.1023221 25790844

[B59] Sahin L. Figueiro M. G. (2013). Alerting effects of short-wavelength (blue) and long-wavelength (red) lights in the afternoon. Physiol. Behav. 116–117, 1–7. 10.1016/j.physbeh.2013.03.014 23535242

[B60] Samuels C. James L. Lawson D. Meeuwisse W. (2016). The athlete sleep screening questionnaire: A new tool for assessing and managing sleep in elite athletes. Br. J. Sports Med. 50, 418–422. 10.1136/bjsports-2014-094332 26002952

[B61] Sander B. Markvart J. Kessel L. Argyraki A. Johnsen K. (2015). Can sleep quality and wellbeing be improved by changing the indoor lighting in the homes of healthy, elderly citizens? Chronobiol. Int. 32, 1049–1060. 10.3109/07420528.2015.1056304 26181467 PMC4673571

[B62] Scheuermaier K. Münch M. Ronda J. M. Duffy J. F. (2018). Improved cognitive morning performance in healthy older adults following blue-enriched light exposure on the previous evening. Behav. Brain Res. 348, 267–275. 10.1016/j.bbr.2018.04.021 29684473 PMC6124504

[B63] Slama H. Deliens G. Schmitz R. Peigneux P. Leproult R. (2015). Afternoon nap and bright light exposure improve cognitive flexibility post lunch. PloS One 10, e0125359. 10.1371/journal.pone.0125359 26016658 PMC4446306

[B64] Spriet L. L. (2014). Exercise and sport performance with low doses of caffeine. Sports Med. 44 (Suppl. 2), S175–S184. 10.1007/s40279-014-0257-8 25355191 PMC4213371

[B65] Srisurapanont K. Samakarn Y. Kamklong B. Siratrairat P. Bumiputra A. Jaikwang M. (2021). Blue-wavelength light therapy for post-traumatic brain injury sleepiness, sleep disturbance, depression, and fatigue: A systematic review and network meta-analysis. PloS One 16, e0246172. 10.1371/journal.pone.0246172 33539446 PMC7861530

[B66] Strong R. E. Marchant B. K. Reimherr F. W. Williams E. Soni P. Mestas R. (2009). Narrow-band blue-light treatment of seasonal affective disorder in adults and the influence of additional nonseasonal symptoms. Depress. Anxiety 26, 273–278. 10.1002/da.20538 19016463

[B67] Stroop J. R. (1935). Studies of interference in serial verbal reactions. J. Exp. Psychol. 18, 643–662. 10.1037/h0054651

[B68] Tähkämö L. Partonen T. Pesonen A.-K. (2019). Systematic review of light exposure impact on human circadian rhythm. Chronobiol. Int. 36, 151–170. 10.1080/07420528.2018.1527773 30311830

[B69] Taillard J. Capelli A. Sagaspe P. Anund A. Akerstedt T. Philip P. (2012). In-car nocturnal blue light exposure improves motorway driving: A randomized controlled trial. PloS One 7, e46750. 10.1371/journal.pone.0046750 23094031 PMC3477137

[B70] Thorpe R. T. Atkinson G. Drust B. Gregson W. (2017). Monitoring fatigue status in elite team-sport athletes: Implications for practice. Int. J. Sports Physiol. Perform. 12, S227–S234. 10.1123/ijspp.2016-0434 28095065

[B71] Tordjman S. Chokron S. Delorme R. Charrier A. Bellissant E. Jaafari N. (2017). Melatonin: Pharmacology, functions and therapeutic benefits. Curr. Neuropharmacol. 15, 434–443. 10.2174/1570159X14666161228122115 28503116 PMC5405617

[B72] Tulppo M. P. Jurvelin H. Roivainen E. Nissilä J. Hautala A. J. Kiviniemi A. M. (2014). Effects of bright light treatment on psychomotor speed in athletes. Front. Physiol. 5, 184. 10.3389/fphys.2014.00184 24860513 PMC4026757

[B73] Van Cutsem J. Marcora S. De Pauw K. Bailey S. Meeusen R. Roelands B. (2017). The effects of mental fatigue on physical performance: A systematic review. Sports Med. 47, 1569–1588. 10.1007/s40279-016-0672-0 28044281

[B74] Van Der Lely S. Frey S. Garbazza C. Wirz-Justice A. Jenni O. G. Steiner R. (2015). Blue blocker glasses as a countermeasure for alerting effects of evening light-emitting diode screen exposure in male teenagers. J. Adolesc. Health 56, 113–119. 10.1016/j.jadohealth.2014.08.002 25287985

[B75] Vandewalle G. Collignon O. Hull J. T. Daneault V. Albouy G. Lepore F. (2013). Blue light stimulates cognitive brain activity in visually blind individuals. J. Cogn. Neurosci. 25, 2072–2085. 10.1162/jocn_a_00450 23859643 PMC4497579

[B76] Viola A. U. James L. M. Schlangen L. J. M. Dijk D.-J. (2008). Blue-enriched white light in the workplace improves self-reported alertness, performance and sleep quality. Scand. J. Work Environ. Health 34, 297–306. 10.5271/sjweh.1268 18815716

[B77] Yang M. Ma N. Zhu Y. Su Y.-C. Chen Q. Hsiao F.-C. (2018). The acute effects of intermittent light exposure in the evening on alertness and subsequent sleep architecture. Int. J. Environ. Res. Public Health 15, 524. 10.3390/ijerph15030524 PMC5877069 29543731

[B78] Zimmermann P. Fimm B. (2002). “A test battery for attentional performance,” in Applied neuropsychology of attention (Herzogenrath: Psychology Press).

